# Манифестация эндокринной офтальмопатии после химиотерапии бендамустином и ритуксимабом у пациента с хроническим лимфолейкозом: клинический случай и обзор литературы

**DOI:** 10.14341/probl13616

**Published:** 2026-05-20

**Authors:** Е. Д. Козлов, Е. Г. Бессмертная, С. Ш. Чандола, Е. И. Ямашкина

**Affiliations:** Мордовский государственный университет имени Н.П. Огарёва; Ogarev Mordovia State University; Научный медицинский исследовательский центр эндокринологии им. академика И.И. Дедова; Endocrinology Research Centre

**Keywords:** болезнь Грейвса, эндокринная офтальмопатия, бендамустин, ритуксимаб, хронический лимфолейкоз, аутоиммунные заболевания, клинический случай, Graves’ disease, hyperthyroidism, Graves’ orbitopathy, thyroid eye disease, bendamustine, rituximab, chronic lymphocytic leukemia, autoimmunity, case report

## Abstract

Эндокринная офтальмопатия (ЭОП) — это самостоятельное прогрессирующее аутоиммунное заболевание органа зрения, чаще всего ассоциированное с болезнью Грейвса. Несмотря на то, что ритуксимаб применяется off-label при активной стероидорезистентной ЭОП, описаны случаи парадоксальной активации аутоиммунных процессов на фоне его применения. Мы представляем редкий случай манифестации ЭОП после химиотерапии бендамустином и ритуксимабом (BR) по поводу хронического лимфолейкоза (ХЛЛ). У 73-летнего мужчины с 3-летним анамнезом болезни Грейвса через 2 недели после второго цикла BR-химиотерапии появились двусторонний экзофтальм, диплопия и снижение остроты зрения. Пациент не курил, радиойодтерапия не проводилась. При обследовании обнаружены высокие уровни антител к рецептору ТТГ, утолщение глазодвигательных мышц по данным МРТ орбит. Диагностирована активная фаза (CAS 6/6) среднетяжелой ЭОП (EUGOGO). Несмотря на два курса пульс-терапии метилпреднизолоном (суммарная доза — 12,2 г) и 10 ретробульбарных инъекций дексаметазона, заболевание прогрессировало до тяжелой степени, осложненной оптической нейропатией со снижением зрительных функций. Хотя у пациента была предрасположенность к аутоиммунным нарушениям, тесная временная связь с BR-терапией, быстрое прогрессирование и резистентность к глюкокортикоидам указывают на значимую роль химиотерапии в нарушении иммунного гомеостаза. Ритуксимаб-индуцированная деплеция В-клеток и синдром высвобождения цитокинов могли способствовать Т-клеточному воспалению в орбите, а бендамустин — усугубить состояние за счет лимфопении и нарушения иммунной толерантности. Данный клинический случай иллюстрирует потенциальную парадоксальную активацию аутоиммунного заболевания после BR-химиотерапии у предрасположенного пациента. Это определяет необходимость скрининга пациентов с сопутствующим аутоиммунным заболеванием щитовидной железы на наличие ЭОП до, во время и после BR-химиотерапии по поводу ХЛЛ. Необходимы дальнейшие исследования для верификации данного феномена.

## Abstract (en)

Graves’ orbitopathy (GO) is a progressive autoimmune disorder of visual organ, mostly associated with Graves’ disease (GD). Although rituximab has been used as an off-label therapy for steroid-resistant GO, paradoxical immune reactivation has also been reported. We present a rare case of GO that developed after bendamustine-rituximab (BR) therapy for chronic lymphocytic leukemia (CLL). A 73-year-old male with a 3-year history of GD, presented with new onset of bilateral proptosis, diplopia, and decreased visual acuity, that happened two weeks after his second BR cycle for CLL. The anamnesis was negative for both smoking, and radioiodine therapy. Laboratory studies showed persistently elevated thyrotropin receptor antibodies levels in plasma, and extraocular muscle enlargement on orbital MRI. The active phase (CAS 6/6) of moderate-to-severe (EUGOGO) GO was diagnosed. Despite two courses of intravenous methylprednisolone (cumulative dose — 12.2 g) and 10 retrobulbar dexamethasone injections, the patient progressed to dysthyroid optic neuropathy. Although the patient had a predisposing autoimmune background, the close temporal association with BR therapy, progressive disease course, and glucocorticoid resistance suggest that chemotherapy-induced immune dysregulation played a critical role in triggering GO. Rituximab-related B-cell depletion and cytokine release syndrome may have facilitated T-cell–mediated orbital inflammation, while bendamustine-induced lymphopenia further disrupted immune tolerance. This case illustrates the potential for paradoxical autoimmune activation following BR therapy in a predisposed patient. Clinicians should monitor for GO signs, and symptoms before, during, and after BR therapy in patients with underlying thyroid autoimmunity. Further clinical trials are needed to verify this phenomenon.

## ВВЕДЕНИЕ

Эндокринная офтальмопатия (ЭОП; орбитопатия Грейвса (ОГ)) — самостоятельное прогрессирующее аутоиммунное заболевание органа зрения, ассоциированное с патологией щитовидной железы (чаще всего с болезнью Грейвса (БГ)), вызывающее такие симптомы, как экзофтальм, боль и двоение в глазах, ощущение песка в глазах, снижение остроты зрения, и другие [1]. К факторам риска ЭОП относятся женский пол, генетическая предрасположенность, курение, высокие уровни антител к рецепторам тиреотропного гормона (а/т к рТТГ), недостаточный контроль тиреоидной дисфункции, радиойодтерапия (РЙТ) и гиперхолестеринемия. Также известно, что пациенты мужского пола в пожилом возрасте имеют повышенный риск более тяжелого течения ЭОП [2][3][4]. Патогенез ЭОП до конца не изучен, однако современные данные указывают на участие клеточных и гуморальных иммунных механизмов. Воспалительный процесс в глазнице развивается, когда антигенпрезентирующие клетки и В-лимфоциты активируют Т-клетки. Это приводит к высвобождению провоспалительных цитокинов, таких как фактор некроза опухоли альфа (TNF-α), гамма-интерферон (IFN-γ), интерлейкин-1 (IL-1) и интерлейкин-6 (IL-6). Цитокины и а/т к рТТГ, произведенные В-лимфоцитами (CD20+), посредством активации специфических рецепторов, стимулируют дифференцировку орбитальных фибробластов в адипоциты и выработку гликозаминогликанов, включая гиалуроновую кислоту. В результате происходит гипертрофия орбитальных адипоцитов, отек орбитальных мышц и мягких тканей глазницы и развитие других признаков воспаления, дополнительно опосредованных хемокинами Th1 (ось CXCL9-11/CXCR3) [2][5][6]. Течение ЭОП варьирует от легкого, при котором чаще всего достаточно симптоматического лечения, до среднетяжелого, требующего введения высоких доз стероидов (4,5–8,0 г внутривенного метилпреднизолона), и угрожающего зрению, в ряде случаев, требующего хирургической декомпрессии орбиты. Препаратом второй линии терапии ЭОП, помимо других, является ритуксимаб (назначение off-label) [5–7].

Ритуксимаб, представляющий собой химерное моноклональное антитело, направленное против антигена CD20+ на поверхности B-клеток, вызывает немедленное истощение пула B-лимфоцитов через механизмы антителозависимой и комплемент-зависимой цитотоксичности, а также регуляции апоптоза [5][6]. Снижение количества B-клеток приводит к уменьшению их способности к презентации антигенов Т-хэлперам (CD4+) и секреции цитокинов (IL-6, IFN-γ). Это нашло применение в терапии аутоиммунных заболеваний, включая ЭОП, а также онкологических заболеваний, таких как хронический лимфоцитарный лейкоз (ХЛЛ) [5][6][8].

ХЛЛ является одним из наиболее распространенных типов лейкоза у взрослых, чаще встречающимся у мужчин. Заболевание характеризуется клональной пролиферацией зрелых B-клеток (CD5+) в периферической крови, костном мозге, лимфоузлах и селезенке. ХЛЛ протекает медленно, но неизлечим, что требует пожизненной терапии. Благодаря высокой эффективности и благоприятному профилю токсичности комбинация бендамустина и ритуксимаба (BR) является эффективным методом терапии ХЛЛ, особенно у пожилых пациентов и пациентов с сопутствующими заболеваниями. Бендамустин вызывает повреждение ДНК и апоптоз раковых клеток, в то время как ритуксимаб усиливает этот эффект, взаимодействуя с антигеном CD20+ на поверхности B-клеток, что приводит к их лизису через комплемент-зависимую цитотоксичность и антителозависимую клеточную цитотоксичность [8].

В данной работе представлен случай парадоксальной манифестации ЭОП (резистентной к терапии стероидами) после второго цикла химиотерапии бендамустином и ритуксимабом у пациента с ХЛЛ и сопутствующей болезнью Грейвса.

## ОПИСАНИЕ СЛУЧАЯ

В эндокринологическое отделение Мордовской Республиканской клинической больницы №4 6 мая 2024 г. поступил 73-летний мужчина с анамнезом болезни Грейвса, жалобами на двустороннее пучеглазие, двоение в глазах, боль при движении глазных яблок, покраснение конъюнктивы и снижение зрения. Данные симптомы развились впервые, примерно через 2 недели после завершения второго цикла химиотерапии по схеме BR, проведенного 22 марта 2024 г. по поводу ХЛЛ.

Болезнь Грейвса впервые диагностирована в октябре 2020 г. На тот момент лабораторные данные свидетельствовали о низком уровне тиреотропного гормона (ТТГ) (<0,0083 мМЕ/л), повышении свободного трийодтиронина (свT3) (27,4 нмоль/л) и а/т к рТГГ (17,54 МЕ/л). Была назначена терапия тирозолом в стартовой дозе 30 мг/сут. Однако с декабря 2020 г. до января 2023 г. пациент не соблюдал режим терапии (изредка принимал тирозол 5 мг/сут), что привело к неконтролируемому гипертиреозу (рис. 1, табл. 1). В январе 2023 г. вновь инициирована тиреостатическая терапия (тирозол 25 мг/сут). Эутиреоза удалось достичь в феврале 2023 г., а контроля над БГ — к октябрю 2023 г. (поддерживающая доза тирозола — 20 мг/сут) (рис. 1, табл. 1).

**Figure:**
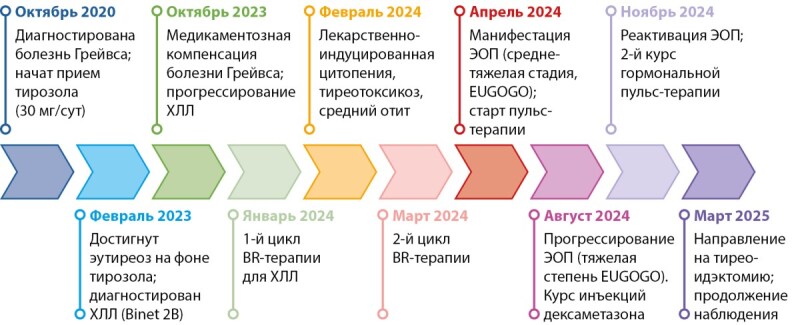
Рисунок 1. Хронология ключевых клинических событий и проводимой терапии. Примечание: ХЛЛ — хронический лимфолейкоз, ЭОП — эндокринная офтальмопатия, BR-терапия — химиотерапия бендамустином и ритуксимабом; EUGOGO — Европейская группа по изучению орбитопатии Грейвса.

**Table:** Таблица 1. Динамика тиреоидных, гематологических и иммунологических параметров у пациента с ХЛЛ и болезнью Грейвса до и после BR-химиотерапии Примечание: АИТ — аутоиммунный тиреоидит, а/т к рТТГ — антитела к рецептору ТТГ, БГ — болезнь Грейвса, ЛУ — лимфатические узлы, Н/И — не исследовалось, Н/П — не применимо, ТТГ — тиреотропный гормон, ХЛЛ — хронический лимфолейкоз, ЩЖ — щитовидная железа, ЭОП — эндокринная офтальмопатия, BR — бендамустин + ритуксимаб, Ds — диагноз.

Показатель / хронология событий	Октябрь 2020(Ds БГ)	Январь 2023 (Ds ХЛЛ)	Октябрь 2023(Пре-BR)	Март 2024(Пост-BR / Дебют ЭОП)	Референсные значения
Тиреограмма
ТТГ(мЕД/л)	<0,0083	<0,0083	0,42	<0,0083	0,4–4,0
свТ4(пмоль/л)	29,91	28,14	11,20	21,57	9,0–19,05
свТ3(пмоль/л)	27,4	14,7	4,8	5,6	3,0–5,6
а/т к рТТГ(МЕ/л)	17,54	Н/И	8,70	10,30	<1,8
Гематологические показатели
Лейкоциты (тыс/мкл)	21,80	21,13	25,94	3,72	4,0–10,0
Лимфоциты (тыс/мкл)	17,29	16,06	21,01	0,80	1,0–4,8
Гемоглобин(г/л)	124	118	120	113	130–170
Тромбоциты (тыс/мкл)	204	180	350	128	150–400
Общий холестерин (ммоль/л)	4,02	4,17	4,34	4,05	<5,2
Цитологическое исследование пунктата костного мозга
		Гипоцеллюлярность гранулоцитарного ростка 25,4%; эритроцитарного ростка 12,2%; мегакариоциты — 5 в 2 мазках; тромбоцитарные клетки собраны в кластеры по 23–46; лимфоцитарный росток увеличен до 59,7%	Лимфоцитарный росток увеличен до 88,8%, из них 6,6% — пролимфоциты; гранулоцитарный и эритроцитарный ростки значительно снижены; мегакариоциты — в достаточном количестве		Н/П
Ультразвуковое исследование ЩЖ
Общий объем, см³	16,4	63	73,9	73,9	<25,0
Отклонения	Эхопризнаки АИТ; изоэхогенный узел правой доли ЩЖ 15x17 мм	Эхопризнаки АИТ; изоэхогенный узел правой доли ЩЖ 15x19 мм	Эхопризнаки АИТ; изоэхогенный узел правой доли ЩЖ 33x19 мм, гиперэхогенный узел левой доли ЩЖ 26x18 мм; ЛУ шеи увеличены до 35x18мм (“гроздья винограда”)	Эхопризнаки АИТ; изоэхогенный узел правой доли ЩЖ 33x19 мм, гиперэхогенный узел левой доли ЩЖ 26x18 мм; ЛУ шеи увеличены до 22 мм	Н/П

ХЛЛ был заподозрен в октябре 2020 г. ввиду выраженного лимфоцитоза (лейкоциты 21 800 клеток/мкл, лимфоциты 17 900 клеток/мкл) и подтвержден в феврале 2023 г. (стадия 2B по классификации Бине) на основании результатов биопсии костного мозга, выявившей выраженную лимфоцитарную инфильтрацию (59,7%) (рис. 1, табл. 1). В октябре 2023 г., по достижении компенсации БГ, у пациента развилась прогрессирующая цервикальная лимфаденопатия. Была выполнена проточная цитометрия, которая подтвердила и иммунотипировала диагноз ХЛЛ (CD5+/CD19+/CD20+/CD22+/CD23+). Пациенту был назначен курс химиотерапии по схеме BR, включающий шесть циклов. Первый цикл химиотерапии был 15 января 2024 г. и привел к улучшению гематологических показателей. Однако вскоре у пациента развилась лекарственно-индуцированная цитопения, а также утрачен контроль над болезнью Грейвса (рис. 1, табл. 1), что потребовало интенсификации тиреостатической терапии (увеличение дозы тирозола до 25 мг/сут на 1 месяц). 19 марта у пациента развился левосторонний средний отит, потребовавший противовоспалительной и антибактериальной терапии. Из-за данных нежелательных явлений второй цикл химиотерапии был отсрочен до 22 марта, однако привел к достижению гематологической ремиссии ХЛЛ (рис. 1, табл. 1).

Наследственный анамнез: у дочери пациента — хронический аутоиммунный тиреоидит с диффузно-узловым зобом. Пациент никогда не курил, не получал препаратов йода, радикальное лечение методом радиойодтерапии (РЙТ) не проводилось. Из сопутствующих заболеваний имеется артериальная гипертензия II стадии, контролируемая приемом бисопролола (5 мг/сут) и зофеноприла (7,5 мг/сут).

При физикальном обследовании у пациента отмечалось нормостеническое телосложение с массой тела 70 кг и ростом 162 см. Частота пульса и сердечных сокращений составляла 83 уд/мин, артериальное давление — 132/81 мм рт.ст. Отмечался легкий интенционный тремор верхних конечностей. Щитовидная железа увеличена до 2‑й степени, плотноэластической консистенции, безболезненная при пальпации.

При офтальмологическом обследовании, согласно классификации EUGOGO (Европейская группа по изучению орбитопатии Грейвса), стадия ЭОП по шкале NOSPECS была классифицирована как средне-тяжелая, активность ЭОП по шкале CAS составила 6/6 баллов (рис. 2 и 3А).

**Figure:**
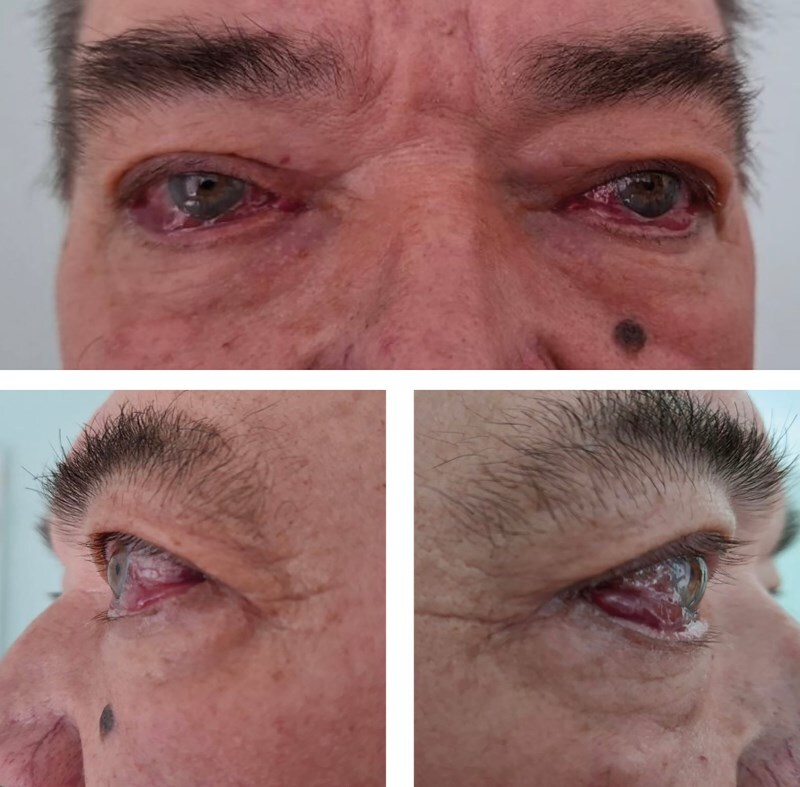
Рисунок 2. Хемоз конъюнктивы и умеренный отек век, развившиеся после второго курса химиотерапии. Положительные глазные симптомы (Штельвага, Дальримпля (OD<OS), Грефе (OD<OS), Кохера (OD<OS), Мёбиуса) (май 2024 г.).

**Figure:**
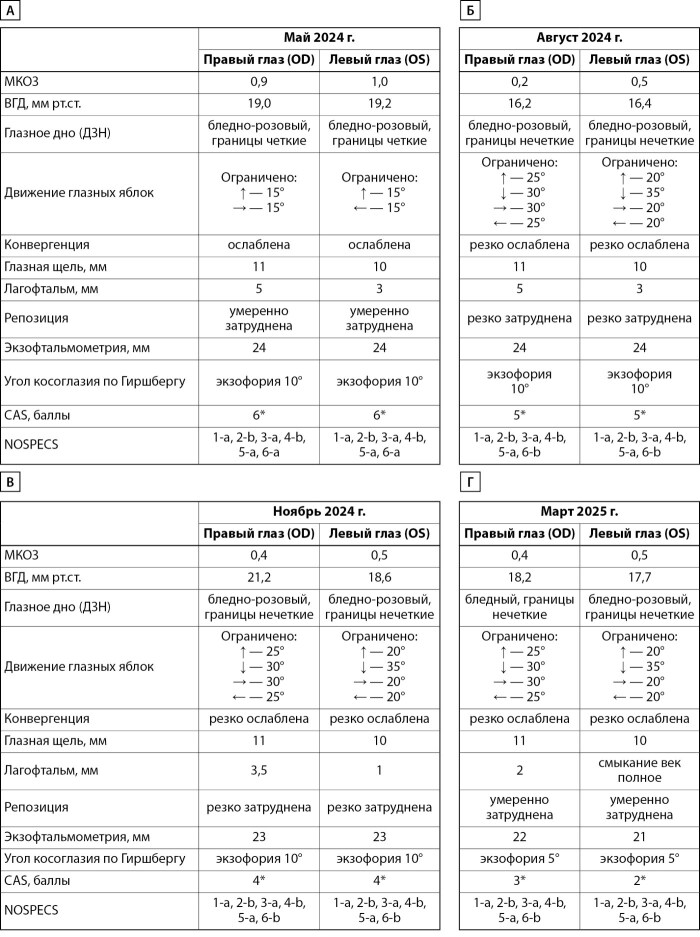
Рисунок 3. Офтальмологический статус пациента в динамике. Примечание: ВГД — внутриглазное давление; ДЗН — диск зрительного нерва; МКОЗ — максимально корригированная острота зрения; ↑ — движение глазных яблок вверх; ↓ — движение глазных яблок вниз; → — движение глазных яблок вправо; ← — движение глазных яблок влево.*Симптомы: периорбитальные отеки, покраснение век, инъекция конъюнктивы, хемоз конъюнктивы, отек слезного мясца, полулунной складки, боли при движении глаз.

Магнитно-резонансная томография (МРТ) орбит и головного мозга от 20.04.2024 г.: глазные яблоки расположены симметрично, их дорсальные поверхности определяются на расстоянии 1 мм кзади от средней межскуловой линии (N=9,9 +/- 1,7 мм [7]). Глазодвигательные мышцы утолщены до 10 мм, отечны, больше слева. Зрительные нервы симметричны, структурны, диаметром 2,5 мм в середине орбиты. Слева, сзади от глазницы, по контуру сифона левой внутренней сонной артерии по ходу интракраниального сегмента левого зрительного нерва, определяется образование неправильной формы, с четкими неровными контурами, имеющее характеристики жировой ткани, размерами 17х15х16 мм.

С мая по сентябрь 2024 г. пациент получал пульс-терапию метилпреднизолоном (курсовая доза — 5,625 г), что привело к временному снижению активности ЭОП. Однако в августе 2024 г. отмечена реактивация ЭОП с прогрессирующим снижением зрения и развитием оптической нейропатии (ОН); по шкале NOSPECS была диагностирована тяжелая степень ЭОП (рис. 1 и 3Б). В связи с чем была рекомендована комбинированная терапия (продолжение пульс-терапии метилпреднизолоном одновременно с лучевой терапией орбит).

Ввиду недоступности лучевой терапии было выполнено 10 ретробульбарных инъекций дексаметазона, что привело к временному уменьшению степени выраженности симптомов ЭОП и незначительному улучшению зрения.

В ноябре 2024 г. возникла реактивация ЭОП (рис. 1, 3В, 4А и 4В). Для дальнейшего обследования и лечения пациент был направлен в НМИЦ эндокринологии, где был рекомендован повторный курс пульс-терапии метилпреднизолоном.

**Figure:**
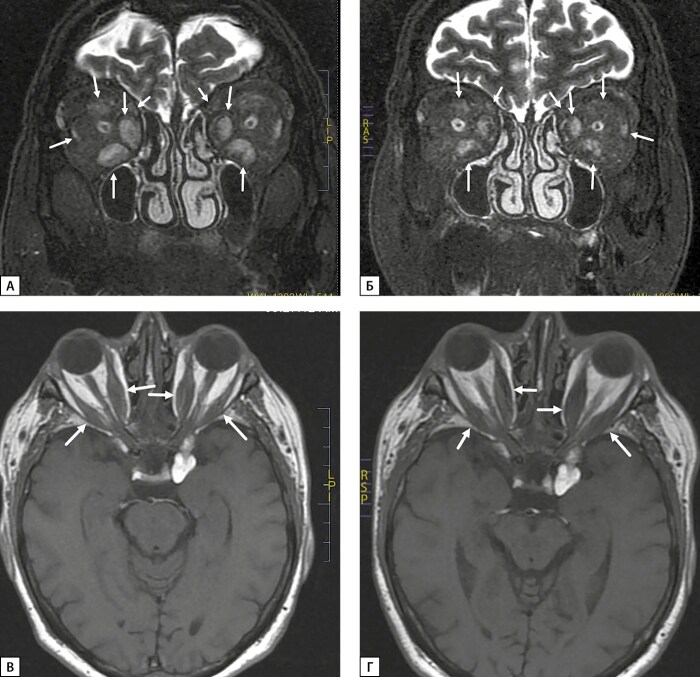
Рисунок 4. МРТ орбит (А, Б — корональная проекция; В, Г — аксиальная проекция) перед вторым курсом пульс-терапии метилпреднизолоном (левые рисунки; ноябрь 2024 г.) и после ее завершения (правые рисунки; март 2025 г.) А. Утолщение и признаки отека ЭОМ, РБК (ЭОМ указаны стрелками).Б. Уменьшение толщины ЭОМ, выраженное уменьшение отека ЭОМ, РБК.В. Утолщение латеральных и медиальных прямых мышц, апикальное сгущение (ЭОМ указаны стрелками).Г. Апикальное сгущение сохраняется, отек ЭОМ менее выражен.

Пульс-терапия была завершена в марте 2025 г., суммарная доза метилпреднизолона составила 12,2 г. В результате проведенного лечения удалось достичь снижения активности ЭОП (CAS 3/2), стабилизации ЭОП (рис. 3Г, 4Б, 4Г и 5) и в марте 2025 г. пациент был направлен на плановую тиреоидэктомию.

**Figure:**
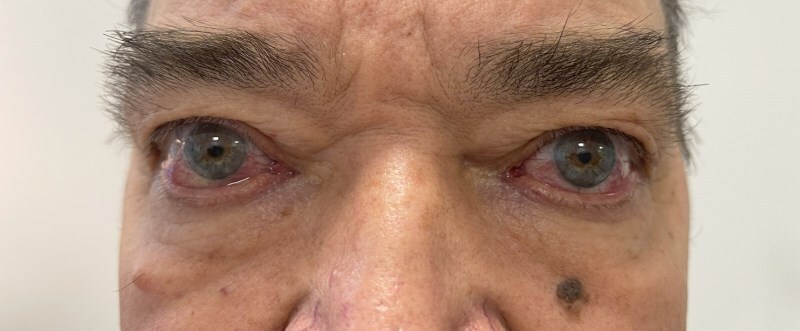
Рисунок 5. Уменьшение проптоза, периорбитальных отеков, инъекции конъюнктивы и хемоза после окончания пульс-терапии метилпреднизолоном (март 2025 г.).

Следует отметить, что пациент сохранял эутиреоидное состояние с февраля 2023 по январь 2024 г., а затем с июня 2024 г. до тиреоидэктомии, что достигалось приемом тиамазола 20 мг/сут. Вместе с тем пациент завершил только 2 из 6 запланированных циклов BR-химиотерапии по поводу хронического лимфоцитарного лейкоза (ХЛЛ) с достижением полного ответа в периферической крови. В настоящее время пациент продолжает находиться под динамическим наблюдением.

## ОБСУЖДЕНИЕ

Насколько нам известно, данный случай манифестации средне-тяжелой, прогрессирующей до тяжелой степени ЭОП, с развитием ОН, после второго цикла BR-терапии по поводу ХЛЛ у пациента с сопутствующей БГ, является первым описанным в литературе. Временная связь между дебютом офтальмопатии и курсом химиотерапии, прогрессирующее течение и резистентность к стандартной гормональной терапии вызывают вопрос о возможной причинно-следственной связи между BR-химиотерапией и активацией аутоиммунного процесса в орбите. Однако нельзя исключить естественное течение ЭОП.

ЭОП является наиболее распространенным экстратиреоидным проявлением болезни Грейвса, встречающимся у 25–50% пациентов [3][4]. У нашего пациента на протяжении всего периода наблюдения сохранялся повышенный уровень а/т к рТТГ (табл. 1) — известного независимого фактора риска ЭОП, а также имеется отягощенный семейный анамнез аутоиммунных тиреопатий, что указывает на генетическую предрасположенность. Несмотря на достижение эутиреоза незадолго до развития офтальмопатии, контроль над БГ оставался неудовлетворительным более двух лет, что также является фактором риска. ЭОП может развиться или прогрессировать спустя месяцы–годы после диагностики БГ (медиана – 18 месяцев), даже на фоне эутиреоза, особенно при персистирующем повышении а/т к рТТГ [2–4][7]. Для прогнозирования ЭОП предложена шкала PREDIGO [9], согласно которой наш пациент имел 8 из 15 баллов до начала BR-терапии, что соответствует умеренному риску ЭОП. Однако из-за низкой прогностической ценности инструмента (0,28) этот результат не позволяет сделать уверенный вывод о возможном развитии заболевания [9].

Стоит отметить, что у 70% пациентов при дебюте БГ по данным МРТ выявляется субклиническое воспаление тканей орбиты, что может предшествовать явной клинической стадии [3]. Поскольку в данном клиническом случае исходная МРТ орбит не проводилась, нельзя исключить «немую» фазу ЭОП до химиотерапии. Однако также известно, что лишь у 5–10% пациентов ЭОП прогрессирует со временем (при отсутствии тяжелых форм в дебюте), тогда как у большинства на фоне тиреостатической терапии наблюдается ремиссия или регресс клинических проявлений [10][11].

Тем не менее отсутствие глазных симптомов в течение трех лет у некурящего пациента с 1-летней историей эутиреоза на фоне тиреостатической терапии и внезапное развитие прогрессирующей, резистентной к стероидам ЭОП через 2 недели после BR-химиотерапии (рис. 1) свидетельствуют о более сложном иммунологическом взаимодействии, чем просто естественное течение заболевания.

Ритуксимаб (химерное моноклональное антитело к CD20+ B-лимфоцитам) вызывает глубокую, но временную B-клеточную деплецию за счет антителозависимой цитотоксичности, активации комплемента и апоптоза [5][6]. Это свойство позволяет применять препарат off-label при активной стероид-резистентной ЭОП, с вариабельной эффективностью [5–7]. Парадоксально, но ритуксимаб ассоциирован с индукцией/обострением аутоиммунных заболеваний, включая системную красную волчанку, ревматоидный артрит и БГ, а также с обострением симптомов ЭОП [12–14].

Механистически ритуксимаб истощает регуляторные B-клетки, в норме поддерживающие иммунную толерантность. Их потеря вместе с нарушением процесса презентации антигенов могут привести к неконтролируемому ответу аутореактивных T-клеток и провоспалительному цитокиновому каскаду, особенно в фазе иммунного восстановления, когда наивные и аутореактивные B-клеточные клоны репопулируют [6][12]. Кроме того, долгоживущие плазматические клетки, устойчивые к ритуксимабу, продолжают продуцировать патогенные а/т к рТТГ (что имеет место у нашего пациента), поддерживая активность заболевания несмотря на B-клеточную деплецию [6].

Бендамустин (алкилирующий агент с цитотоксическим и иммуносупрессивным действием) дополнительно нарушает иммунный гомеостаз. Он вызывает глубокую T- и B-клеточную лимфопению и подавляет регуляторные T-клетки, создавая временный «иммунный вакуум», благоприятный для аутоиммунной активации [15][16]. В нашем случае лимфоциты пациента снизились до уровня 0,8×10⁹/л после химиотерапии (табл. 1), что согласуется с гипотезой синдрома иммунной реконституции.

Провоспалительные сигналы (IL-6, растворимый рецептор IL-6, CXCL10), вовлеченные в патогенез ЭОП, недостаточно подавляются ритуксимабом [17]. Бендамустин, в свою очередь, может способствовать поляризации иммунного ответа в сторону Th1/Th17 и активации сигнального пути IGF-1R/IL-6/STAT3, что приводит к резистентности к стероидной терапии и персистенции воспалительного процесса в орбите.

Несмотря на редкость, в литературе описаны случаи, подтверждающие возможность развития или обострения ЭОП на фоне химиотерапии. N. Mora et al. (2019) описали случай возникновения БГ после R-CHOP терапии у пациента с диффузной В-крупноклеточной лимфомой и сопутствующим тиреоидитом Хашимото, связав это с B-клеточной реконституцией [13]. C. Liu et al. (2018) сообщили о случае обострения течения ранее существовавшей ЭОП после каждого цикла R-CHOP терапии у пациента с В-клеточной лимфомой, успешно контролируемого назначением глюкокортикоидов, однако с негативным исходом [14]. Парадоксально, но ухудшение глазной симптоматики (хемоз, снижение зрения, дистиреоидная оптическая нейропатия) также документировано среди пациентов получающих ритуксимаб по поводу ЭОП [18–21]. Данный феномен может быть объяснен синдромом высвобождения цитокинов или прямой иммунотоксичностью препарата [14][18–21]. Кроме того, имеются данные о возможном развитии ЭОП на фоне терапии ингибиторами иммунных контрольных точек (ремелимумаб, дурвалумаб) и алемтузумабом [22][23]. Таким образом, иммуномодулирующая терапия может провоцировать/обострять ЭОП посредством дисрегуляции иммунного гомеостаза, активации аутореактивных T-клеток, сдвига цитокинового баланса в провоспалительную сторону.

Уникальность данного случая обусловлена: (1) четкой временной связью манифестации ЭОП со 2-м циклом BR терапии, (2) резистентностью к пульс-терапии глюкокортикоидами (рефрактерная форма ЭОП), (3) прогрессирующим течением с развитием ОН, что потребовало интенсификации гормональной пульс-терапии.

Подобное агрессивное течение при отсутствии традиционных триггеров (РЙТ, анамнез курения) указывает на ключевую роль BR-терапии в патогенезе. Наиболее вероятно, что BR-индуцированная иммунная дерегуляция «разрешила» манифестацию латентного аутоиммунного процесса у генетически предрасположенного пациента с персистирующими а/т к рТТГ.

## ЗАКЛЮЧЕНИЕ

Данный клинический случай иллюстрирует сложное взаимодействие между иммунотерапией онкологического заболевания и эндокринным аутоиммунитетом. Химиотерапия ритуксимабом и бендамустином, несмотря на эффективность при лимфопролиферативных заболеваниях, может парадоксально индуцировать/усугублять ЭОП у предрасположенных лиц. Для верификации и более глубокого понимания патофизиологии данного феномена необходимы дальнейшие исследования. Клиницисты должны проявлять особую настороженность в отношении проявлений ЭОП у пациентов с болезнью Грейвса, получающих BR-химиотерапию по поводу ХЛЛ, даже при эутиреоидном статусе. Профилактические меры могут предусматривать стратификацию риска ЭОП на основе шкал, таких как PREDIGO, проведение базовой офтальмологической оценки, мониторинг офтальмологического статуса, МРТ орбит по показаниям и мониторинг уровня а/т к рТТГ. Перспективные исследования также могут быть направлены на поиск прогностических биомаркеров (таких как антитела к IGF-1R, IL-6), а также на разработку таргетной терапии, минимизирующей аутоиммунные риски без ущерба для противоопухолевой эффективности. Этот случай подчеркивает необходимость междисциплинарного подхода к ведению пациентов с сочетанной онкологической и аутоиммунной патологией, требующего сотрудничества между онкологами, эндокринологами, офтальмологами и иммунологами.

## ДОПОЛНИТЕЛЬНАЯ ИНФОРМАЦИЯ

Источники финансирования. Работа выполнена по инициативе авторов без привлечения финансирования.

Конфликт интересов. Авторы декларируют отсутствие явных и потенциальных конфликтов интересов, связанных с содержанием настоящей статьи.

Участие авторов. Авторы декларируют соответствие своего авторства международным критериям ICMJE. Все авторы внесли равный вклад в подготовку статьи, одобрили финальную версию статьи перед публикацией, выразили согласие нести ответственность за все аспекты работы, подразумевающую надлежащее изучение и решение вопросов, связанных с точностью или добросовестностью любой части работы.

Согласие пациента. Пациент добровольно подписал информированное согласие на публикацию персональной медицинской информации в обезличенной форме в журнале «Проблемы эндокринологии».
